# Sleep enhances reconsolidation-based strengthening of visuospatial memories

**DOI:** 10.1038/s41598-022-11135-6

**Published:** 2022-05-04

**Authors:** Bethany J. Jones, Margaret E. Chen, Lindsey Simoncini, Rebecca M. C. Spencer

**Affiliations:** 1 Department of Psychological & Brain Sciences, University of Massachusetts, Amherst, MA 01003 USA; 2 Neuroscience & Behavior Program, University of Massachusetts, Amherst, MA 01003 USA; 3 Commonwealth Honors College, University of Massachusetts, Amherst, MA 01003 USA; 4 Institute for Applied Life Sciences, University of Massachusetts, 240 Thatcher Way, S315, Amherst, MA 01003 USA

**Keywords:** Consolidation, Long-term memory, Neurophysiology, Human behaviour

## Abstract

Consolidated memories can be returned to a labile state upon reactivation. The re-stabilization of reactivated memories, or reconsolidation, can allow for change in previously established memories. Given the role of sleep in the initial consolidation of memories, sleep may be important for reconsolidation as well. However, effects of sleep on reconsolidation and specific aspects of sleep that may contribute are unclear. Here, participants learned 30 picture-location pairs. After overnight sleep, initial consolidation was tested. Following either one day (Experiment 1) or one week (Experiment 2), participants were tested again to reactivate their memory and then learned 30 novel picture-location pairs. Control groups (Experiment 1) received no reactivation prior to new learning. Twelve hours later, after daytime wakefulness or overnight sleep, participants completed a final memory test. Sleep participants underwent polysomnography between reactivation and final tests. In Experiment 1, reactivation led to preservation of memory compared to no reactivation. Sleep was associated with less post-reactivation memory decline than waking, with memory preservation positively related to time spent in non-rapid-eye movement sleep. In Experiment 2, sleep was associated with greater post-reactivation memory improvement than waking, with improvement positively related to sigma activity. These results suggest sleep enhances reconsolidation-based strengthening of episodic memories.

## Introduction

Following learning, memories undergo an initial consolidation process that transforms the labile, short-term representation into a more stable, long-term one. Sleep has been widely implicated in this process^1,2^. In particular, temporally-aligned interactions among cortical slow waves, thalamocortical spindles, and hippocampal sharp-wave ripples during non-rapid eye movement (NREM) sleep are thought to underlie consolidation^3,4^. During this process, hippocampal memory traces are replayed in order to strengthen corresponding neocortical representations.

Despite relative stability, consolidated memories can be returned to a labile state, in which they are vulnerable to modification, via reactivation during wake. Reactivated memories are re-stabilized via *reconsolidation*, and interventions targeting this process can lead to weakening, strengthening, or updating of memories^5,6^. For example, protein synthesis inhibitors block reconsolidation and weaken memory^7,8^, whereas mild stress and exercise enhance reconsolidation and strengthen memory^9–11^. Notably, memory lability and reconsolidation are more reliably triggered when new learning is present or there is prediction error, consistent with a role in memory updating^12–14^. Novel learning following reactivation can lead to memory interference and/or addition of new information into the reactivated memory^15–20^, whereas relearning the same material after reactivation can strengthen the reactivated memory^21,22^. As reconsolidation allows for memory change, it may facilitate remediation of maladaptive memories^23^.

Initial studies demonstrated that reconsolidation takes time, with effects typically observed days following reactivation but not immediately or shortly after^8,17,20^. Given this timeline, together with the role of sleep in the initial consolidation of memories, sleep has been presumed to be important for reconsolidation as well^24^. In rodents, sleep deprivation following memory reactivation impairs subsequent memory performance, suggesting this may indeed be the case^25–27^. In humans, post-reactivation memory performance has been linked to features of intervening NREM sleep^28–30^. In particular, Bryant et al.^30^ found that sleep spindle density during NREM2 sleep the night after memory reactivation predicted updating of the memory with new information. However, in previous studies, the influence of sleep was not shown to be specific to reactivated memories^28,29^, and/or the influence of reactivation was not shown to be specific to sleep^29,30^. To our knowledge, only one study has shown post-reactivation memory change that was dependent on both reactivation and sleep. Specifically, Kindt and Soeter^31^ found that post-reactivation beta-adrenergic receptor blockade reduced fear memory when followed by 12 h of sleep opportunity but not 12 h of wakefulness. However, this study lacked objective measures of sleep. Thus, the influence of sleep on reconsolidation, and the particular features of sleep potentially involved in this process, warrant further investigation.

**Figure 1:**
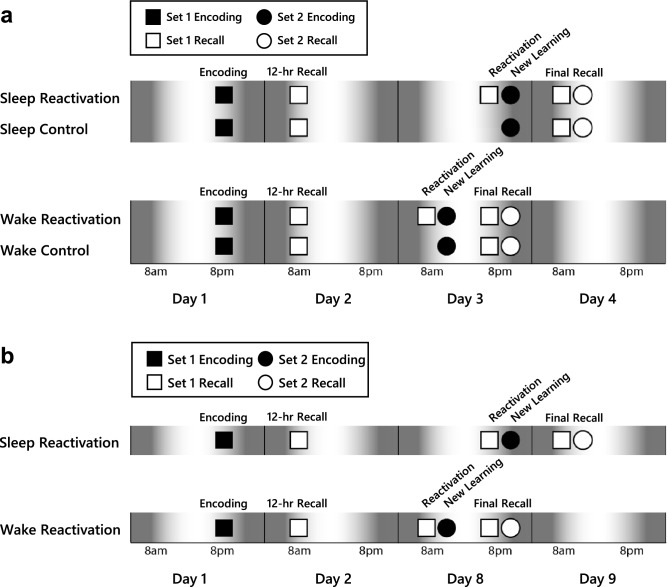
Experimental timeline and procedure. (**a**) In Experiment 1, participants encoded Set 1 in the evening of day 1 and were tested on Set 1 the morning of day 2 (12-h recall). On day 3 in the morning (wake group) or the evening (sleep group), participants in the reactivation groups were tested on Set 1 and then encoded Set 2, whereas participants in the control groups only encoded Set 2. Twelve hours later, participants were given a final test first on Set 1, then on Set 2 (final recall). (**b**) In Experiment 2, participants encoded Set 1 in the evening of day 1 and were tested on Set 1 the morning of day 2 (12-h recall). On day 8 in the morning (wake group) or the evening (sleep group), participants were tested on Set 1 prior to encoding Set 2. Twelve hours later, participants were given a final test first on Set 1, then on Set 2 (final recall).

In the present study we manipulated both memory reactivation status and sleep status in order to determine the effect of sleep on reconsolidation. Furthermore, we measured overnight sleep with polysomnography (PSG) to determine which features of sleep may be associated with reconsolidation. Our primary hypothesis was that sleep would enhance reconsolidation-based memory change. Based on previous studies^15,19,20^, we expected that reactivating a consolidated memory prior to new learning would lead to more forgetting/interference of the original memory compared to not reactivating the original memory prior to new learning. Thus, we hypothesized that post-reactivation memory weakening would be greater over a period of sleep than wake. Given the link between NREM sleep and memory consolidation, we further hypothesized that features of NREM sleep would be associated with post-reactivation memory reduction. However, if interference does not occur, reconsolidation could lead to memory strengthening^32,33^, in which case we would hypothesize that sleep would be linked to greater strengthening, and NREM sleep features would predict post-reactivation memory improvement or preservation. We first tested our hypotheses in an experiment where memory was reactivated after a relatively short retention interval (2 days from learning; Experiment 1). Because memory performance was very strong at the time of reactivation, which could have influenced the outcome, we sought to further test our hypotheses in a second experiment where memory was reactivated after a longer retention interval (1 week from learning) that allowed for more forgetting prior to reactivation (Experiment 2; Fig. 1).

## Results

**Figure 2:**
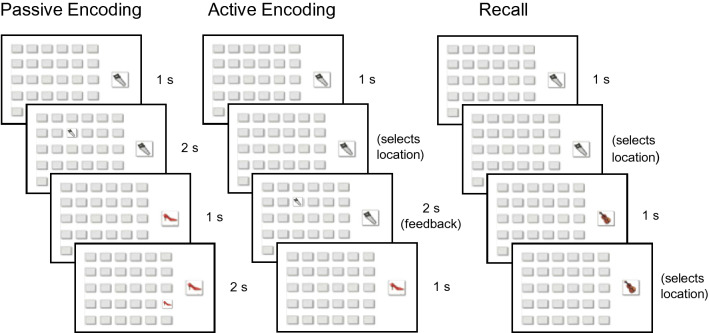
Visuospatial task. During passive encoding, participants were shown the locations of 30 pictures. During active encoding, participants selected a location for each picture and were shown the correct location as feedback. During recall, participants selected a location for each picture and were not provided feedback.

Reconsolidation was induced by reactivating memory for locations of one set of pictures (Set 1) prior to encoding locations of a new set of pictures (Set 2; see Fig. 2 for depiction of the visuospatial task). As illustrated in Fig. 1, Set 1 encoding took place on the evening of day 1, followed by 3 recall sessions: (1) 12-h recall, which took place the next morning and was included to assess initial consolidation; (2) reactivation recall, which took place either 2 days (Experiment 1) or 7 days (Experiment 2) after encoding, in either the morning (wake groups) or evening (sleep groups), and served to both reactivate the memory and assess performance in the reactivation groups (control groups did not undergo reactivation recall); and (3) final recall, which took place 12 h after reactivation recall. Set 2 encoding (new learning) took place on day 3 (Experiment 1) or day 8 (Experiment 2), immediately after reactivation recall of Set 1 in the reactivation groups. Recall of Set 2 took place 12 h after Set 2 encoding (final recall session).

### Reactivation leads to Set 1 memory strengthening (Experiment 1)

Set 1 learning and memory performance is presented in Table 1. We first sought to assess whether the reactivation procedure evoked reconsolidation by comparing reactivation and control groups in Experiment 1. Though potential reconsolidation in the reactivation groups would have occurred after the reactivation session (between reactivation recall and final recall), we assessed the effect of reactivation over the longer interval between 12-h recall and final recall, because the control groups had no reactivation recall test. A 2 × 2 ANOVA with reactivation group and sleep group as between-subjects factors indicated a main effect of reactivation group (*F*(1,91) = 28.533, *p* < 0.001, partial η^2^ = 0.239), with less memory decline in the reactivation groups than control groups. There was no main effect of sleep group (*p* = 0.912) and no interaction between group factors (*p* = 0.823).

**Table 1:** Set 1 learning and memory performance (mean (*SE*)).

	*N*	# Encoding rounds	Encoding recall (%)	12-h recall (%)	Reactivation recall (%)	Final recall (%)
**Experiment 1**						
Sleep reactivation	23	4.35 (0.42)	88.91 (0.99)	86.52 (1.06)	83.04 (1.73)	83.77 (1.61)
Sleep control	23	4.39 (0.42)	86.52 (1.68)	87.83 (2.22)	–	75.51 (3.22)
Wake reactivation	26	4.81 (0.44)	89.53 (1.03)	91.54 (1.20)	90.51 (1.43)	88.59 (1.57)
Wake control	23	4.91 (0.50)	87.39 (0.96)	88.26 (1.45)	–	76.52 (2.53)
**Experiment 2**						
Sleep reactivation	19	4.68 (0.48)	88.25 (0.82)	88.42 (1.45)	64.39 (2.92)	73.33 (3.18)
Wake reactivation	17	5.0 (0.67)	89.02 (2.23)	87.45 (3.25)	69.80 (4.94)	70.39 (4.71)

These results suggest that reactivation led to strengthening (preservation) of memory when followed by either sleep or wakefulness. Though contrary to our initial expectation that reactivation would lead to memory weakening due to interference from new learning, this result is consistent with accounts of reconsolidation-based memory strengthening^32,33^. Sleep did not interact with reactivation status when considering memory change between 12-h recall and final recall, possibly because this time interval included not only the potential reconsolidation window (post-reactivation) but also time between 12-h recall and reactivation recall, which differed between wake and sleep groups (24 h vs. 36 h). Thus, an effect of sleep on reconsolidation could have been obscured by sleep/wake differences prior to reactivation in this analysis.

### Sleep enhances reconsolidation-based strengthening

After determining that the overall effect of reactivation was memory strengthening, we sought to more closely assess the role of sleep in reconsolidation-based strengthening by comparing post-reactivation memory change between sleep and wake reactivation groups. Characteristics of post-reactivation sleep are presented in Table 2.

**Table 2:** Post-reactivation sleep characteristics (mean (*SE*)).

	Experiment 1	Experiment 2
TST (min)	411.22 (11.56)	413.04 (12.24)
SL (min)	14.31 (2.36)	29.06 (6.97)
SE (%)	91.66 (1.47)	91.28 (1.52)
RL (min)	82.16 (6.38)	96.66 (10.26)
NREM1 (%)	5.12 (0.33)	5.33 (0.38)
NREM2 (%)	57.32 (1.28)	55.19 (1.10)
SWS (%)	16.44 (1.27)	18.5 (1.30)
REM (%)	21.36 (0.95)	21.01 (1.16)
Delta density	20.75 (1.00)	19.33 (0.91)
Sigma density	3.16 (0.16)	2.90 (0.13)

*TST* total sleep time, *SL* sleep latency, *SE* sleep efficiency, *RL* REM latency.

#### Experiment 1

**Figure 3:**
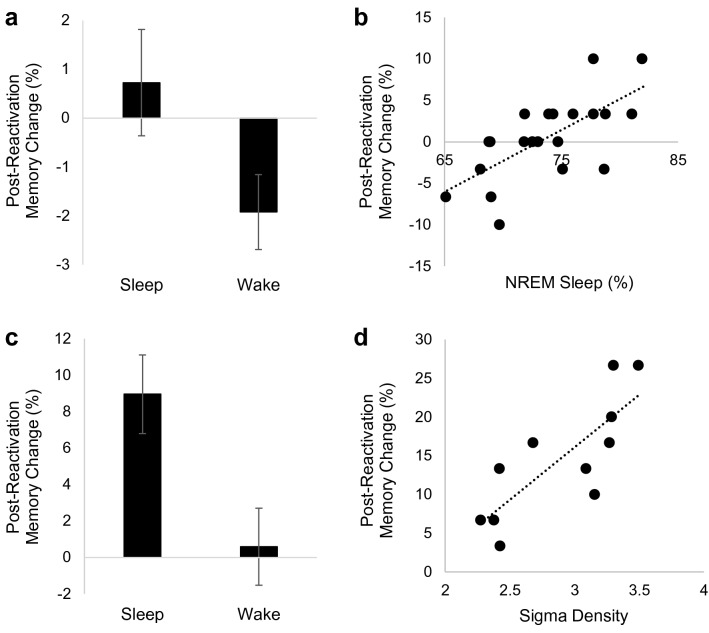
Effect of sleep on reconsolidation-based memory strengthening. (**a**) Average Set 1 memory change between reactivation recall and final recall in Experiment 1. (**b**) Relationship between time in NREM sleep (NREM2 and SWS) and Set 1 memory change between reactivation recall and final recall in the sleep group in Experiment 1. (**c**) Average Set 1 memory change between reactivation recall and final recall in Experiment 2. (**d**) Relationship between NREM sigma density and Set 1 memory change between reactivation recall and final recall in the sleep group in Experiment 2. Error bars represent standard errors of means.

In Experiment 1, a 2 × 2 mixed ANOVA with sleep group as a between-subjects factor and session (reactivation recall, final recall) as a within-subjects factor indicated a main effect of sleep group (*F*(1,47) = 8.225, *p* = 0.006, partial η^2^ = 0.149), with higher memory accuracy in the wake group, no main effect of session (*p* = 0.364), and a sleep group X session interaction (*F*(1,47) = 4.10, *p* = 0.049, partial η^2^ = 0.08). Follow up paired-samples *t*-tests indicated significant memory decline in the wake group (*t* = 2.51, *p* = 0.019, *d* = 0.49) and no change (preservation) in the sleep group (*t* = – 0.666, *p* = 0.512; Fig. 3A). Memory preservation in the sleep reactivation group was positively associated with percent time spent in NREM2 (*r* = 0.46, *p* = 0.041) and percent time in NREM (NREM2 and SWS combined; *r* = 0.69, *p* = 0.001; Fig. 3B). Relationships with other sleep variables (SWS%, delta activity, sigma activity) were not significant (*p*’s > 0.24).

#### Experiment 2

In Experiment 2, a 2 × 2 mixed ANOVA with sleep group as a between-subjects factor and session (reactivation recall, final recall) as a within-subjects factor indicated a main effect of session (*F*(1,34) = 9.883, *p* = 0.003, partial η^2^ = 0.225), no main effect of sleep group (*p* = 0.819), and a sleep group X session interaction (*F*(1,34) = 7.595, *p* = 0.009, partial η^2^ = 0.183). Follow up paired-samples *t*-tests indicated significant memory improvement in the sleep group (*t* = − 4.135, *p* = 0.001, *d* = 0.95) and no change in the wake group (*p* = 0.784; Fig. 3C). There was a significant positive relationship between sigma density and memory change between reactivation recall and final recall in the sleep group (*r* = 0.79, *p* = 0.004; Fig. 3D). Relationships with other sleep variables were not significant (*p*’s > 0.42). Together, these results suggest that sleep, and particularly NREM sleep mechanisms, benefit reconsolidation-based memory strengthening.

### Effects of sleep are not explained by differences in encoding, consolidation, or reactivation

#### Experiment 1

Neither the number of encoding rounds needed to reach the accuracy criterion (*p* = 0.75), nor the accuracy during the final round of encoding (*p* = 0.26) differed among groups, suggesting equivalent memory encoding of Set 1. Regarding Set 1 consolidation, there was marginal evidence that the overnight change in memory between encoding recall and 12-h recall differed among the four groups (*F*(3,91) = 2.43, *p* = 0.070, η^2^ = 0.07), with post-hoc pairwise comparisons providing marginal evidence for more overnight decline in accuracy in the sleep reactivation group than in the wake reactivation group (*p* = 0.052, *d* = 0.81; no differences between other group pairs, *p*’s > 0.20). Given that group procedures did not differ at this point, this result is unexpected but suggests initial consolidation of Set 1 may have been stronger in the wake reactivation group than the sleep reactivation group. However, initial consolidation was not related to post-reactivation memory change in either group (*p*’s > 0.35), suggesting that a potential difference in consolidation did not influence reconsolidation.

Regarding memory reactivation, the wake reactivation group had higher memory accuracy during reactivation recall than did the sleep reactivation group (*t* = 3.36, *p* = 0.002, *d* = 0.96), perhaps reflecting initial consolidation differences and/or time since encoding (36 h in the wake group vs. 48 h in the sleep group). Reactivation recall accuracy was not related to subsequent memory change in the wake group (*p* = 0.83). However, there was a significant negative relationship in the sleep group (*r* = − 0.42, *p* = 0.049), indicating that better performance at reactivation recall was associated with less subsequent improvement, perhaps due to ceiling effects. Thus, though there was no relationship in the wake group, it is possible that ceiling effects limited the ability to detect further improvement more so in the wake group than sleep group, and/or reconsolidation may have a larger effect on weaker memories^34,35^.

#### Experiment 2

As in Experiment 1, neither the number of encoding rounds needed to reach the accuracy criterion (*p* = 0.70), nor the accuracy during the final round of encoding (*p* = 0.74) differed between groups, suggesting equivalent memory encoding of Set 1. Likewise, regarding consolidation of Set 1, overnight change in accuracy between encoding and 12-h recall did not differ between sleep and wake reactivation groups (*p* = 0.43), suggesting equivalent initial consolidation of Set 1. With regard to reactivation, there was no difference between sleep and wake groups on reactivation recall accuracy (*p* = 0.34), suggesting equivalent memory reactivation strength. Notably, unlike in Experiment 1, reactivation recall accuracy was well below ceiling, and thus there was adequate room to observe subsequent improvement in both groups.

Taken together, results of these experiments suggest that greater post-reactivation memory preservation/improvement over sleep cannot be explained by group differences in encoding, initial consolidation, or reactivation.

### Encoding and consolidation of set 2

Set 2 learning and memory performance is presented in Table 3.

**Table 3:** Set 2 learning and memory performance (mean (*SE*)).

	*N*	# encoding rounds	Encoding recall (%)	Final recall (%)
**Experiment 1**				
Sleep reactivation	27	3.22 (0.28)	89.01 (0.71)	86.54 (1.44)
Sleep control	24	3.50 (0.44)	90.71 (0.90)	85.14 (1.68)
Wake reactivation	27	2.96 (0.40)	88.52 (0.91)	80.83 (1.45)
Wake control	19	3.32 (0.41)	89.82 (1.18)	68.07 (3.08)
**Experiment 2**				
Sleep reactivation	20	2.85 (0.33)	88.50 (1.15)	84.17 (2.25)
Wake reactivation	17	3.47 (0.52)	83.92 (3.08)	69.61 (4.10)

#### Experiment 1

**Figure 4:**
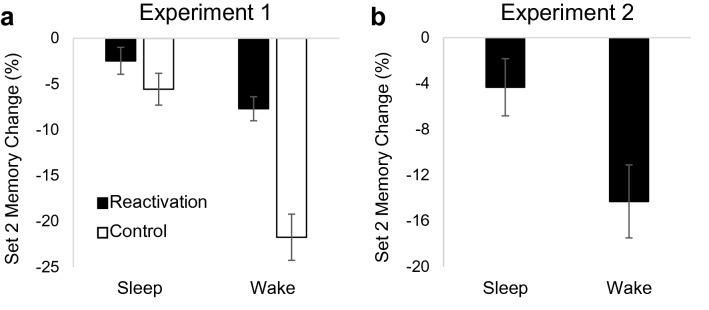
Consolidation of new learning. Average Set 2 memory change between encoding and final recall in Experiment 1 (**a**) and Experiment 2 (**b**). Error bars represent standard errors of means.

Groups did not differ on number of encoding rounds or encoding recall accuracy (*p*’s > 0.33), suggesting equivalent learning among groups. The change in Set 2 memory accuracy between encoding recall and final recall (i.e. consolidation) was compared among groups using a 2 X 2 ANOVA with experimental group (reactivation vs. control) and sleep group (sleep vs. wake) as between-subjects factors. Unsurprisingly, sleep benefitted consolidation of Set 2 (main effect of sleep group: *F*(1,93) = 38.28, *p* < 0.001, partial η^2^ = 0.29; Fig. 4A). Reactivation of Set 1 also benefitted consolidation of Set 2 (main effect of experimental group: *F*(1,93) = 24.64, *p* < 0.001, partial η^2^ = 0.21), and sleep group modulated this effect (sleep group X experimental group interaction: *F*(1,93) = 10.04, *p* = 0.002, partial η^2^ = 0.10). Follow-up testing indicated that reactivation benefitted Set 2 consolidation over wake (*t* = 4.94, *p* < 0.001, *d* = 1.48) but not sleep (*t* = 1.367, *p* = 0.178), likely due to ceiling effects over sleep. There were no significant relationships between NREM sleep variables and Set 2 consolidation (all *p*’s > 0.40).

#### Experiment 2

As in Experiment 1, groups did not differ on number of encoding rounds or encoding recall accuracy (*p*’s > 0.14), suggesting equivalent learning among groups. Again, sleep benefitted consolidation of Set 2, as there was greater preservation of memory between encoding recall and final recall in the sleep reactivation group compared to the wake reactivation group (*t* = 2.50, *p* = 0.017, *d* = 0.82; Fig. 4B). Percent time spent in NREM sleep was positively associated with consolidation of Set 2 (*r* = 0.53, *p* = 0.040). No other relationships were significant (*p*’s > 0.11).

Together, results of these experiments suggest consolidation of Set 2 occurred in parallel with reconsolidation of Set 1 and that reactivation/reconsolidation of prior learning may boost consolidation of similar new learning.

### Subjective sleepiness

Subjective sleepiness was measured at the beginning of each session using the Stanford Sleepiness Scale.

#### Experiment 1

There were no group differences in sleepiness at encoding, 12-h recall, or final recall (all *p*’s > 0.2). At reactivation recall, the wake reactivation group (*M* = 3.03, *SD* = 1.20) was sleepier than the sleep reactivation group (*M* = 2.34, *SD* = 1.21), suggesting more sleepiness in the morning than evening (*t* = − 2.113, *p* = 0.04). Despite greater sleepiness, we note that the wake reactivation group performed better than the sleep reactivation group on Set 1 reactivation recall, and the groups performed equally well on Set 2 encoding. Thus, it seems unlikely that the group difference in sleepiness affected memory outcomes.

#### Experiment 2

There was no difference between groups on subjective sleepiness at any session (all *p*’s > 0.3).

## General discussion

The current study provides evidence that sleep benefits reconsolidation of episodic memories. In Experiment 1, we established that memory reactivation, compared to no reactivation, led to preserved memory. In both experiments, we found that post-reactivation memory preservation/strengthening was greater over a night of sleep than a day of wakefulness. This strengthening was related to time in NREM sleep (Experiment 1) and NREM sigma activity (Experiment 2).

We included new learning following memory reactivation, as previous evidence suggests this may be needed to trigger reconsolidation^13^. New learning can interfere with reconsolidation of the initial memory, leading to memory impairment^15,19,20^. However, in the current study, we observed strengthening of the initial memory. The fact that new learning did not directly conflict with the original memory, as was the case in some previous studies^15,20^, may have allowed for strengthening rather than interference. Additionally, using a test as the reminder protects from retroactive interference compared to a weaker/indirect reminder^36^, and may allow for better memory segregation^37^. Reconsolidation also leads to strengthening when reactivation is followed by re-learning of the same material^21,22^, mild stress^9,10^, exercise^11^, or pharmacological interventions^38^. Reactivations in and of themselves have also been observed to strengthen memory^32,33^.

Sleep benefited post-retrieval memory strengthening. Specifically, strengthening was associated with time in NREM sleep and NREM sigma activity. Sigma activity is a proxy for sleep spindle activity (though they are not exactly the same). As such, this finding is consistent with a recent study linking sleep spindles to reactivation-induced memory updating^30^. Sleep spindles are involved in hippocampal-neocortical dialogue, whereby replay of newly encoded memories in the hippocampus aligns with spindle events to promote strengthening of neocortical memory traces^3,39^. Thus, reconsolidation may invoke the same or similar hippocampal-neocortical interactions as initial consolidation. Alternatively, reconsolidation may not engage the full hippocampal-neocortical dialogue but simply induce re-processing of the previously established neocortical representation (without hippocampal reactivation), and sleep spindle activity could index this local re-processing. Notably, there is evidence that reactivation can return memories to a hippocampus-dependent state, perhaps suggesting that hippocampal replay would be involved in systems-level reconsolidation^40^. Given the small samples sizes in our sleep physiology analyses, further research is needed to confirm the role of sleep spindles and otherwise determine the sleep mechanisms involved in reconsolidation-based memory change.

Though sleep boosted strengthening, an effect of reactivation was also observed over wake (Experiment 1), suggesting that sleep may optimize reconsolidation but not be necessary for this process. This is in contrast to previous evidence that sleep may be required to enact reactivation-induced memory weakening. Kindt and Soeter (2018) blocked beta-adrenergic receptors following reactivation of fear memory. Memory impairment was observed after 12 h of sleep but not 12 h of wakefulness, and this effect was selective for the reactivated memory (compared to memory that was not reactivated). Based on the synaptic tagging and capture hypothesis^41^, the authors concluded that blocking beta-adrenergic receptors prevented protein synthesis needed to re-stabilize reactivated/tagged synapses, which led to subsequent synaptic downscaling/weakening during sleep.

Results of our study can likewise be explained by a synaptic tagging and capture mechanism. Memory retrieval destabilized the memory, placing synapses in a tagged state. New learning triggered cellular consolidation mechanisms, including plasticity-related products that were captured by tagged synapses, thus enabling plasticity of not only the new memory but the original memory as well. Re-stabilization preserved memory over periods of wake and sleep compared to no reactivation/re-stabilization, but sleep promoted further strengthening via spindle-associated processing. Thus, as with initial consolidation, sleep may be a fundamental step in reconsolidation of memories, providing processing beyond that which takes place during waking.

Finally, we observed a beneficial effect of reactivation on consolidation of new learning. Memory testing can lead to proactive facilitation by enhancing list segregation^37^ and/or integration of competing information^42^. Memory for changes between stimulus lists is associated with proactive facilitation^43^. Thus, memory retrieval of Set 1 just prior to learning Set 2 may have promoted memory for the changes between the sets, which could have aided consolidation. The effect was only observed in the wake group, presumably because of ceiling effects in the sleep group. As proactive facilitation is typically studied at short retention intervals, more research is needed to understand the process as it relates to consolidation of long-term memory.

In conclusion, results of these experiments suggest that sleep is involved in reconsolidation-based memory strengthening. However, limitations of this study should be considered. First, we used a high learning accuracy criterion (80%), which led to strong memories and made it difficult to detect further strengthening in Experiment 1. Importantly, adding a week to allow for forgetting in Experiment 2 enabled a more robust measurement of post-reactivation memory strengthening. A second limitation is that, because reactivation needed to occur at different times of day in the sleep and wake groups in order to precede naturally occurring sleep and wakefulness, the retention interval between learning and reactivation was 12 h longer in the sleep group than wake group. This difference could have influenced the strength of the reactivated memory and therefore subsequent reconsolidation. Indeed, memory performance/strength at reactivation was not well matched between sleep and wake groups in Experiment 1, which could be attributable to the differing retention interval and/or a circadian effect on task performance. In Experiment 2, adding several more days between learning and reactivation should have diminished the impact of 12 extra hours of retention in the sleep group, since most forgetting would be expected to occur closer to learning. Indeed, reactivation performance was better matched between groups in Experiment 2. Nonetheless, future research using a nap design is needed to completely rule out effects of retention interval and circadian timing.

Another consideration is that the reactivation session took place in different locations for sleep and wake groups. This was done to facilitate in-home PSG recording in the sleep groups but may have led to stronger reactivation in the wake groups, since wake participants’ reactivation session occurred in the same spatial context (our lab) as initial learning, and the spatial context is a particularly effective reminder cue^44^. Notably, we do not see a larger effect of reactivation in the wake group compared to the sleep group, suggesting that if context effects were present they were relatively small compared to the main reactivation effect driven by the recall test. Nonetheless, direct and indirect reminders may have different consequences^36^, and so future studies controlling for spatial context are warranted. Finally, our sample sizes in sleep physiology analyses were somewhat low, particularly with regard to Experiment 2, and samples were unbalanced with regard to gender (majority female). The fact that we see converging results across experiments may offset some concern of low sample size. However, we may have been underpowered to detect relationships with some of the sleep variables we investigated, and these results should be interpreted with caution.

## Method

### Ethics declarations

Experimental procedures were approved by the University of Massachusetts, Amherst Institutional Review Board and were in accordance with the Declaration of Helsinki. Written informed consent was obtained from participants before they began the experiment.

### Participants

112 young adults between 18 and 27 years of age participated in Experiment 1 in one of four groups: sleep reactivation group (n = 28, M = 20.21 year, SD = 1.66 year, 20 females), sleep control group (n = 25, M = 19.67 year, SD = 1.31 year, 16 females), wake reactivation group (n = 33, M = 19.78 year, SD = 1.31 year, 23 females), or wake control group (n = 26, M = 20.31 year, SD = 2.20 year, 19 females). 41 young adults between 18 and 22 years of age participated in Experiment 2 in either a sleep reactivation group (n = 22, M = 19.36 year, SD = 1.00 year, 18 females) or wake reactivation group (n = 19, M = 19.47 year, SD = 1.07 year, 18 females). Participants had normal or corrected-to-normal vision and no history of neurological disease, sleep disorders, head injury, or use of medications known to affect sleep or cognitive function. Participants were instructed to refrain from alcohol and limit caffeine intake during the study. All participants were compensated with payment or course credit.

### Materials

Stimuli were 60 clip art images of common objects. They were split into two separate sets of 30 images each that were matched for object category (animal, food, person, tool, etc.). Images were presented on a computer monitor using MATLAB software. Self-reported sleep duration was assessed using an abbreviated version of the Pittsburg Sleep Diary^45^. Self-reported sleepiness was assessed using the Stanford Sleepiness Scale^46^.

### Procedure

The experiment took place over four sessions: encoding, 12-h recall, reactivation/new learning, and final recall (Fig. 1). Sessions took place over three (wake groups) or four (sleep groups) days, which were either consecutive (Experiment 1; Fig. 1A) or included a 6-day interval between the 12-h recall and reactivation/new learning sessions (Experiment 2; Fig. 1B). The reactivation/new learning session for the sleep groups was conducted in the participants’ homes to facilitate sleep recordings (sleep reactivation groups) and for the purpose of consistency (sleep control group). All other sessions were conducted in the lab.

Participants performed the *encoding session* in the evening of day 1. The task was a computerized 2-D visuospatial task similar to the game “Memory” or “Concentration”, following the design of Sonni & Spencer^47^. The participant was first shown an image on its own for 1 s and then shown that image in its correct location in a 5 × 6 grid of 30 possible locations for 2 s (Fig. 2, Passive Encoding). Image-location pairs were shown one at a time until the participant was shown all 30 pairs (Set 1) in a random order, twice. Next, the participant was shown one of the images on its own and asked to choose the corresponding location (Fig. 2, Active Encoding). Regardless of whether the choice was correct or incorrect, the participant was shown the image in its correct location for 2 s. This test phase continued with the set of 30 images being repeated in loops until the participant reached a criterion of at least 80% correct. Once the task was completed, the participant was sent home and encouraged to have a normal night’s sleep and return the following morning.

The *12-h recall session* took place on the morning of day 2 and was meant to test the memory of Set 1 after a night of initial consolidation. The participant was asked to fill out a sleep–wake diary and then take a recall test. In the recall test, they were shown each of the 30 images once and instructed to choose the correct location in the grid (Fig. 2, Recall). They were not given any feedback regarding whether or not their choice was correct.

The *reactivation/new learning session* took place on day 3 (Experiment 1) or day 8 (Experiment 2) in either the morning (wake groups) or evening (sleep groups). Participants in the reactivation groups, but not the control groups, were first tested on recall of Set 1 (reactivation recall). The purpose of this test was to reactivate the memory of Set 1 and also provide a measurement of memory accuracy at this time. This test was identical to that given in the 12-h recall session. Next, all participants performed the same encoding task as during the first session, but with novel images (Set 2). As before, the task continued until participants reached the criterion of at least 80% correct. Following the new learning task, participants in the sleep reactivation groups underwent application of electrodes for PSG.

Lastly, the *final recall session* occurred on the evening of day 3 (Experiment 1) or day 8 (Experiment 2) for the wake groups and on the morning of day 4 (Experiment 1) or day 9 (Experiment 2) for the sleep groups. All participants were first tested on recall of Set 1, and then following a 5-min break, recall of Set 2. These tests were identical to the recall tests given in previous sessions.

### Polysomnography

PSG (sleep reactivation groups only) was recorded in participants’ homes using the Aura PSG ambulatory system (Grass Technologies). Electrodes applied included two electrooculography (EOG; right and left ocular canthi), two chin electromyography, and seven electroencephalography (EEG; F3, F4, C3, Cz, C4, O1, O2) leads. PSG data were collected at a sampling rate of 200 Hz with a bandpass of 0.1 to 100 Hz. EOG and EEG channels were referenced to Cz during recording and re-referenced to the contralateral mastoid for scoring. Recordings were obtained and scored according to the specifications provided by the American Academy of Sleep Medicine^48^.

### Data analysis

Eight participants (four in Experiment 1, four in Experiment 2) were excluded from all analyses for sleeping less than 5 h following encoding (applicable to both sleep and wake groups) or reactivation/new learning (applicable to sleep groups only) Four additional participants in Experiment 1 were excluded from all analyses due to data loss that affected both Set 1 and Set 2 analyses. Some participants had data loss that affected only Set 1 or Set 2 analyses, in which case they were removed only from the respective analyses (see final sample sizes for these analyses in Tables 1 and 3). Six participants in the sleep reactivation groups (two in Experiment 1, four in Experiment 2) were excluded from all PSG analyses due to recording failure or poor quality, and some additional participants were excluded from analyses of delta (one in Experiment 1, four in Experiment 2) and/or sigma (two in Experiment 1, four in Experiment 2) activity due to poor signal quality in at least one channel used in those analyses. Thus, in Experiment 1, 25 participants were included in sleep stage analysis, 24 in delta activity analysis, and 23 in sigma activity analysis. In Experiment 2, 15 participants were included in sleep stage analysis, and 11 participants were included in delta and sigma activity analyses.

*Memory accuracy* was calculated as the percentage of picture locations correctly identified. *Memory consolidation* was calculated by subtracting the accuracy during the final round of encoding (encoding recall) from the accuracy at 12-h recall (Set 1), or from the accuracy at final recall (Set 2). *Memory reconsolidation* was assessed by subtracting accuracy at reactivation recall from accuracy at final recall.

PSG analyses were conducted in MATLAB using a combination of EEGLAB (Delorme & Makeig, 2004), ERPLAB (Lopez-Calderon & Luck, 2014), and in-house software (PSGpower; https://osf.io/qsryf/). EEG amplitude density was measured in the delta (0.5–4 Hz) and sigma (12–16 Hz) bands over frontal (F3, F4; delta) and central (C3, C4; sigma) scalp regions by extracting the amplitude envelope of bandpass-filtered EEG, summing it within NREM2 and SWS stages, and dividing by the number of samples. A more detailed description of this preprocessing and quantification has been reported previously^49^.

Statistical analyses were conducted in SPSS. Comparisons of means were conducted using analyses of variance (ANOVAs) when comparing among four groups (Experiment 1) and Student’s independent-samples *t*-tests when comparing between two groups (Experiments 1 and 2). Levene’s test for equality of variances was applied, and adjusted *p*-values are reported when applicable. Tukey’s HSD test was used for post-hoc pairwise comparisons. Pearson’s *r* was used to assess bivariate linear relationships. Multivariate outliers were detected and removed based on a Cook’s Distance greater than 3 *SD* from the mean Cook’s Distance (*n* = 0–1 data point removed per correlation analysis).

## Data Availability

The datasets generated during and/or analyzed during the current study are available from the corresponding author on reasonable request.
